# Using artificial intelligence to identify characteristics associated with clinical and economic outcomes in MASH (FOCUS-MASH)

**DOI:** 10.1177/17562848261462398

**Published:** 2026-07-09

**Authors:** Kamal Kant Mangla, Semiu O. Gbadamosi, Daniel Semeniuta, Jigar Bandaria, Joseph Zabinski, Gary Curhan, Costas Boussios

**Affiliations:** Novo Nordisk Service Centre India Pvt. Ltd., Bengaluru, KA 560066, India; Novo Nordisk Inc., Plainsboro, NJ, USA; OM1, Inc., Boston, MA, USA; OM1, Inc., Boston, MA, USA; OM1, Inc., Boston, MA, USA; OM1, Inc., Boston, MA, USA; OM1, Inc., Boston, MA, USA

**Keywords:** fibrosis, liver, nonalcoholic fatty liver disease, nonalcoholic steatohepatitis

## Abstract

**Background:**

Heterogeneity in the disease characteristics of metabolic dysfunction-associated steatohepatitis (MASH) complicates efforts to identify individuals with high unmet need.

**Objective:**

We used artificial intelligence (AI) phenotyping to identify factors associated with rapid fibrosis progression, long-term clinical outcomes, and high healthcare costs.

**Design:**

In this retrospective cohort study (January 1, 2013–September 1, 2022), patients with a MASH diagnosis, a Fibrosis-4 index score within 90 days of first MASH diagnosis, and no other liver diseases were identified in a United States electronic medical records and claims dataset.

**Methods:**

Using machine learning, characteristics, including diagnoses, procedures, and medications, were grouped into phenotypic signals, which were iteratively refined and evaluated for strength of association with each outcome. Differences between subgroups in the proportions of patients with specific phenotypic signals were calculated.

**Results:**

In total, 14,707 patients were included in at least one analysis. Rapid fibrosis progression (*n* = 1795) was associated with a history of anemia, thrombocytopenia, and cardiovascular (CV) diagnoses. Long-term liver- and CV-related outcomes (*n* = 13,880) were associated with chronic diseases, kidney diagnoses, cardiac diagnoses, medications, lab tests, and injuries. High healthcare costs (*n* = 10,133) were associated with heart failure procedure codes, cardiac diagnoses and testing, hospitalization procedure codes and kidney diagnoses, and gastrointestinal diagnoses and abdominal imaging.

**Conclusion:**

This study demonstrates the application of AI phenotyping to multidimensional real-world data. This approach could support new methods for proactively identifying patients in clinical practice who require closer monitoring and management.

## Plain language summary

Study using artificial intelligence to identify factors linked with worse medical outcomes and higher costs in patients with a liver disease called metabolic dysfunction-associated steatohepatitis (MASH)

Metabolic dysfunction-associated steatohepatitis (MASH) can lead to serious conditions like liver scarring and liver failure, but differences between patients make it difficult to predict who is at risk of rapidly progressive disease and severe long-term outcomes and who should therefore be prioritized for early monitoring or treatment. We used an artificial intelligence-based technique called digital phenotyping to analyze medical records and claims data, and found factors, including diagnoses of heart and kidney problems, that were associated with rapid disease progression, long-term clinical outcomes, and higher medical costs. The techniques used could help to create tools for better patient monitoring and management in the future.

## Introduction

Metabolic dysfunction-associated steatotic liver disease (MASLD; previously termed nonalcoholic fatty liver disease (NAFLD)^1^) is the most prevalent chronic liver disease, affecting 30% of adults worldwide.^2^ Of these, approximately 25% develop metabolic dysfunction-associated steatohepatitis (MASH; previously termed nonalcoholic steatohepatitis (NASH)^1^), in which cell death and inflammation drive the development of fibrosis.^3^

Approximately 20% of individuals with MASLD experience rapid fibrosis progression, although the factors determining this are unclear.^4^ Fibrosis progression in MASLD is associated with an increasing risk of liver-related morbidity, including cirrhosis and hepatocellular carcinoma.^5^ Advanced fibrosis is also linked with cardiovascular disease (CVD), independent of metabolic comorbidities,^6^ and a greater risk of mortality, compared with earlier stages.^7^

The economic burden of MASH on healthcare systems is substantial. Global data indicate that healthcare costs for patients with MASLD are nearly twice as high as those for age-matched controls, and are highest in those with advanced fibrosis and end-stage liver disease.^8^ In the United States alone, direct costs associated with MASLD exceed $100 billion per year, with liver transplantation contributing significantly to these costs.^9,10^

Given the burden of MASH and the importance of early disease identification for clinical decision-making, a means of identifying patients at the greatest risk of experiencing rapid fibrosis progression, developing long-term clinical outcomes, and incurring high healthcare costs is strongly needed. However, efforts to identify at-risk patients are complicated by the multifactorial nature of MASH,^11^ substantial inter-patient heterogeneity,^12^ and the absence of a standard method for predicting disease progression. Although liver biopsy is not required for the clinical management of most individuals with MASLD, guidelines recommend its use when there is diagnostic uncertainty.^13,14^ However, it is expensive, invasive, and not without risks.^15,16^ In recent years, clinical practice has moved toward the use of non-invasive biomarkers, such as the Fibrosis-4 index (FIB-4),^17^ which is the most widely used non-invasive method for estimating the risk of advanced fibrosis.^18^

The aim of this study was to use artificial intelligence (AI) phenotyping to identify factors associated with rapid fibrosis progression (analysis 1), the development of long-term clinical outcomes (analysis 2), and high healthcare costs (analysis 3) in a large real-world cohort of patients with MASH.

## Methods

### Data source and study design

**Figure 1.**
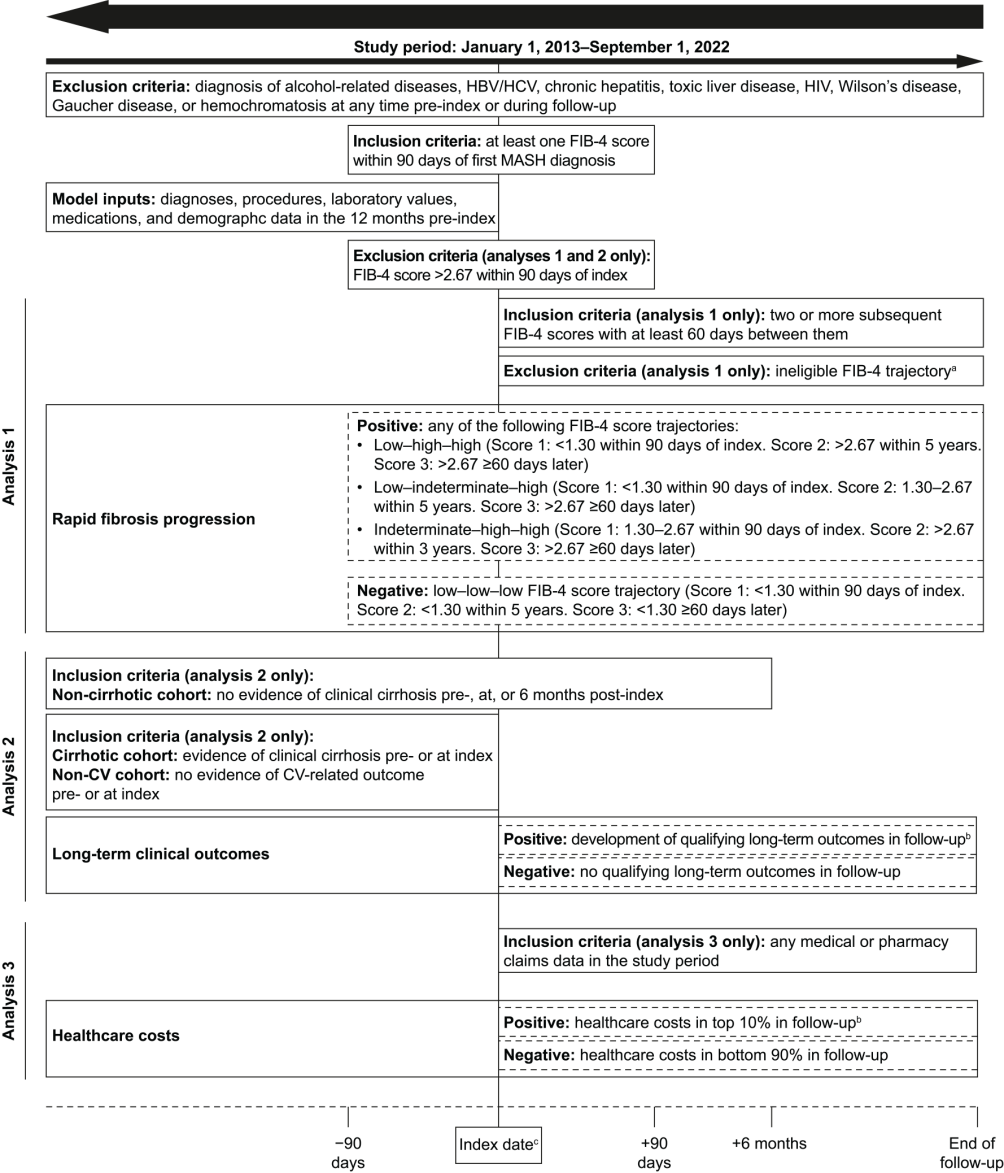
Study design and cohort definitions. ^a^Eligible FIB-4 directories were low–high–high, low–indeterminate–high, indeterminate–high–high, and low–low–low, as detailed in the figure. ^b^Follow-up windows were 5 years and 3 years for those with low and indeterminate FIB-4 scores at baseline, respectively. ^c^The index date for analysis 1 was the first observed FIB-4 score within 90 days of first MASH diagnosis; the index date for analyses 2 and 3 was the date of first MASH diagnosis. CV, cardiovascular; FIB-4, Fibrosis-4 index; HBV, hepatitis B virus; HCV, hepatitis C virus; HIV, human immunodeficiency virus; MASH, metabolic dysfunction-associated steatohepatitis.

This retrospective cohort study used the OM1 Real-World Data Cloud, a multisource dataset derived from United States electronic health records (EHR) and claims data covering more than 340 million people. The OM1 Real-World Data Cloud integrates data from multiple sources, including EHR, medical claims, pharmacy claims, laboratory results, vital signs, and social determinants of health. It encompasses inpatient and outpatient encounters across acute care facilities, ambulatory surgical and medical centers, and specialty clinics. Patients from all 50 United States states and territories are represented. A proprietary patient index links records across data sources while maintaining de-identification. The period of data coverage was January 1, 2013, to September 1, 2022 (Figure 1).

### Selection criteria

Inclusion criteria for all analyses were a diagnosis of MASH and at least one FIB-4 score within 90 days of the first MASH diagnosis (Figure 1). The index date for analysis 1 was the first observed FIB-4 score within 90 days of the first MASH diagnosis; the index date for analyses 2 and 3 was the date of the first MASH diagnosis. Diagnosis of MASH was based on International Classification of Diseases, Tenth Revision, Clinical Modification (ICD-10-CM), MEDCIN, or Systematized Nomenclature of Medicine Clinical Terms (SNOMED CT) diagnosis codes (ICD-10-CM, K75.81; MEDCIN, 313129; SNOMED CT, 442685003).

Exclusion criteria for all analyses were a diagnosis of any of the diseases detailed in Figure 1, either pre-index or during follow-up. Individuals with a FIB-4 score >2.67 within 90 days of first MASH diagnosis were excluded from analysis 1; defining rapid fibrosis progression beyond a high FIB-4 score may not be meaningful because these patients may already be considered for close monitoring and management. These individuals were also excluded from analysis 2. Additional inclusion and exclusion criteria are detailed in Figure 1.

In analysis 2, eligible patients were grouped into cohorts to assess specific clinical outcomes. The non-cirrhotic cohort comprised individuals not diagnosed with cirrhosis, or without evidence of cirrhosis (e.g., esophageal varices), or without a liver-related long-term outcome pre-, at, or 6 months post-index (Figure 1). The cirrhotic cohort comprised individuals diagnosed with cirrhosis and not diagnosed with a liver-related long-term outcome pre- or at index. The non-cardiovascular (CV) cohort comprised individuals not diagnosed with a CV long-term outcome pre- or at index.

### Positive and negative subgroups

Eligible patients were grouped into positive and negative subgroups based on the presence or absence of specific outcomes during follow-up.

In analysis 1, individuals were grouped into fibrosis progression trajectories based on FIB-4 scores over time (score 1 was within 90 days of index, score 2 was within 5 years, and score 3 was ⩾60 days after score 2). Thresholds corresponded to low, indeterminate, and high fibrotic states.^19^ These trajectories aimed to capture the essence of rapid progression within the context of the available data, and in the absence of a standard definition for rapid fibrosis progression. The positive subgroup comprised individuals with the following rapid trajectories: low–low–high, low–indeterminate–high, and indeterminate–high–high. The negative subgroup comprised those without rapid fibrosis progression (low–low–low trajectory).

In analysis 2, individuals were considered positive for long-term clinical outcomes if any of the following were observed during follow-up based on qualifying ICD-9, ICD-10, or SNOMED CT codes: cirrhosis (compensated or decompensated; in the non-cirrhotic cohort only), other liver-related outcomes (liver carcinoma, liver failure, or liver transplantation; in the non-cirrhotic and cirrhotic cohorts), or CV outcomes (heart failure, myocardial infarction, stroke, or unstable angina; in the non-CV cohort only).

In analysis 3, the positive subgroup comprised individuals with annual healthcare costs in the top 10% when averaged over the follow-up period, with all other individuals in the negative subgroup. Healthcare costs were derived from reported cost data encompassing encounter-level and medication costs, from a payer perspective. These data are appropriate for relative comparisons between patient subgroups but do not represent accurate absolute costs.

### Statistical analysis

The AI-based PhenOM™ platform (OM1, Inc., Boston, MA, USA) was calibrated to isolate phenotypic signals associated with each outcome. Inputs included diagnoses, procedures, laboratory values, medications, and demographic data in the 12 months pre-index.

An overview of the modeling process is shown in the Supplemental Figure. The calibration process first relied on the PhenOM™ platform’s AI-based generation of groupings of thematically related data elements (e.g., grouping a set of diagnoses, procedures, and medications together), which fully covered each patient’s medical record. This process uses carefully designed self-supervised algorithms that operate on each patient’s medical history and unsupervised algorithms that produce the final groupings. Although clinicians typically craft these groupings, PhenOM™ produces the first version automatically. That version is essentially ready for final validation, requiring only a brief expert review to confirm the strength of outcome associations and ensure clinical plausibility. By automating the creation of these thematically related groupings, the AI algorithm eliminates the painstaking manual effort that would otherwise be necessary. Furthermore, supervised learning could potentially result in overfitting and the need for quantitative statistical validation against external datasets.

Phenotypic signals were given descriptive thematic labels after their isolation, and they were not constructed or amended based on these labels. Although multiple phenotypic signals may have similar descriptive labels, individual data elements can appear only within a single signal. Phenotypic signals underwent directional significance testing, comprising bootstrapped resampling of the positive and negative subgroups across 1000 iterations in each instance, and subsequent calculation of “directionality” (i.e., overrepresentation in the positive cohort or in the negative cohort). Phenotypic signals passing this testing showed the same directionality at least 95% of the time, indicating stable performance in distinguishing positive and negative subgroups.

Phenotypic signals were assessed using alpha and beta metrics. Alpha metrics were calculated by subtracting proportions in the negative subgroups from proportions in the positive subgroups. Beta metrics were calculated by dividing the greater proportion by the lesser (regardless of subgroup). Alpha metrics reveal coarser large-scale differences between subgroups, whereas beta metrics reveal finer differences and can identify populations with distinct characteristics within subgroups. Higher values indicate a phenotypic signal’s importance in distinguishing subgroups. For example, a phenotypic signal present in 40% of the positive subgroup and 10% of the negative subgroup would have an alpha value of 0.3 and a beta value of 4.0.

The PhenOM™ platform was used here to quantitatively characterize phenotypic signals associated with each outcome at the population level. The findings generate hypotheses that can be further explored in rigorous confirmatory epidemiological studies, as well as provide a foundation for future predictive modeling work; they do not, however, constitute a predictive model, and the alpha and beta metrics reported should not be interpreted as predictive scores applicable to individual patients.

### Reporting guidelines

This study is reported in accordance with the Strengthening the Reporting of Observational Studies in Epidemiology (STROBE) guidelines.^20^

## Results

### Study attrition

**Figure 2.**
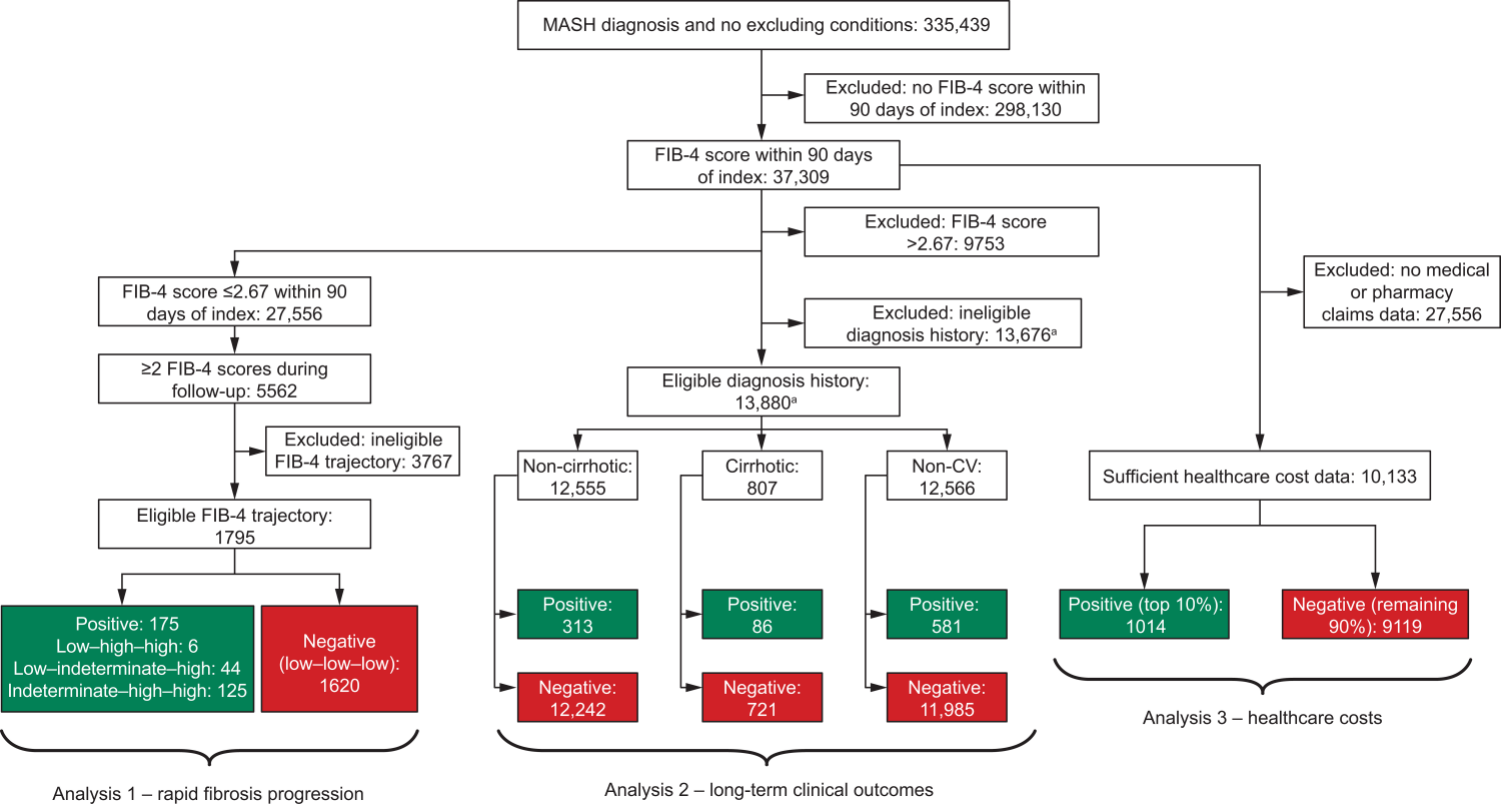
Study attrition. ^a^Eligible diagnosis histories differed between cohorts: no evidence of clinical cirrhosis pre-, at, or 6 months post-index (non-cirrhotic cohort), evidence of clinical cirrhosis pre- or at index (cirrhotic cohort), no evidence of a CV-related outcome pre- or at index (non-CV cohort). Some patients are featured in more than one cohort, so the total is lower than the sum of the totals for each cohort. CV, cardiovascular; FIB-4, Fibrosis-4 index; MASH, metabolic dysfunction-associated steatohepatitis.

Of 474,602 individuals with a MASH diagnosis in the dataset, 335,439 had no excluding conditions. Following the application of eligibility criteria, 14,707 individuals were included in at least one analysis. Full details of study attrition are provided in Figure 2.

### Patient characteristics

In analysis 1, patients with rapid fibrosis progression trajectories (positive subgroup) were older, on average, than patients without (negative subgroup; 66.3 vs 54.6 years; Supplemental Table). Age was a relatively strong factor in the initial results distinguishing the subgroups (and is included in the calculation of FIB-4), so subsequent analyses for analysis 1 included only patients >40 years old.

Similarly, in analyses 2 and 3, patients with long-term clinical outcomes or with healthcare costs in the top 10% (positive subgroups) were older, on average, than patients in the respective negative subgroups (non-cirrhotic cohort: 57.9 vs 53.3 years; cirrhotic cohort: 62.0 vs 59.8 years; non-CV cohort: 61.7 vs 52.9 years; healthcare costs cohort: 58.4 vs 54.2 years).

### Rapid fibrosis progression analysis (analysis 1)

**Figure 3.**
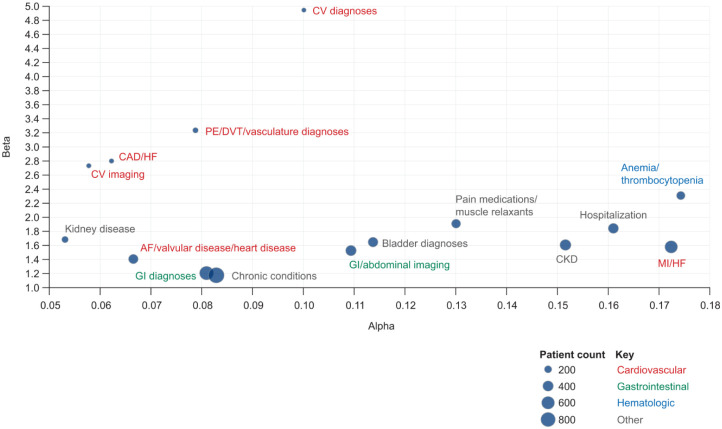
Analysis 1—Key themes associated with rapid fibrosis progression. Alpha and beta metrics represent, respectively, the absolute and relative differences between subgroups in the proportions of individuals with each phenotypic signal. Phenotypic signals with higher alpha and/or beta values are more closely linked with the outcome of interest. AF, atrial fibrillation; CAD, coronary artery disease; CKD, chronic kidney disease; CV, cardiovascular; DVT, deep vein thrombosis; GI, gastrointestinal; HF, heart failure; MI, myocardial infarction; PE, pulmonary embolism.

The top theme for rapid fibrosis progression (*n* = 1795), based on the alpha metric, was a history of diagnoses of anemia and thrombocytopenia, including general anemia, iron deficiency anemia, and general thrombocytopenia (Figure 3). These themes were observed in association with procedures relating to the diagnosis and management of these conditions, such as blood smears. Another key theme was a history of heart failure and related procedures. The elements of this theme included prothrombin time, electrocardiogram, emergency room visits, partial thromboplastin time, and troponin. In some patients, these were combined with procedures related to infection and sepsis. Patients with a history of hospitalization (including initial and subsequent hospital care) were also at elevated risk for rapid fibrosis progression. Kidney diagnoses emerged as a primary theme in combination with hospitalization, including acute kidney failure, chronic kidney disease (CKD), and hypokalemia.

The top theme, based on the beta metric, was CV diagnoses, including atrial fibrillation, dilated cardiomyopathy, coronary artery disease, myocardial infarction, and congestive heart failure (Figure 3). Other major themes included pulmonary embolism and deep vein thrombosis. These CV diagnoses were also reflected in the myocardial infarction/heart failure theme.

### Long-term clinical outcomes analysis (analysis 2)

Given the smaller cohort size for this analysis, phenotypic signals were included only after meeting the criteria for directional significance testing in 95% of samples and a minimum of 10% prevalence in the respective positive subgroup.

### Long-term liver-related outcomes, including cirrhosis

**Figure 4.**
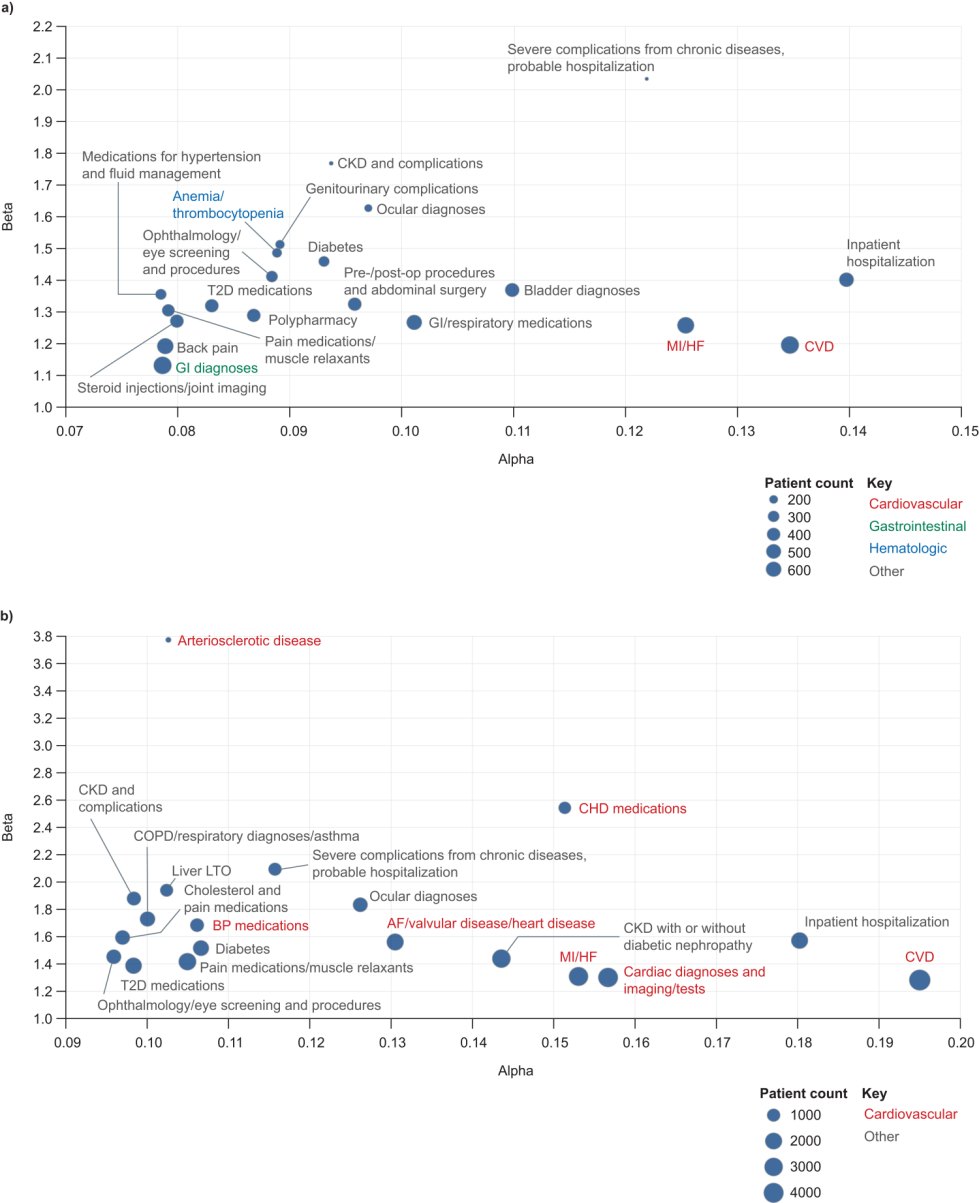
Analysis 2—Key themes associated with (a) long-term liver-related clinical outcomes, including cirrhosis (non-cirrhotic cohort) and (b) long-term CV-related outcomes (non-CV cohort). Alpha and beta metrics represent the absolute and relative differences between subgroups, respectively, in the proportions of individuals with each phenotypic signal. Phenotypic signals with higher alpha and/or beta values are more closely linked with the outcome of interest. AF, atrial fibrillation; BP, blood pressure; CHD, coronary heart disease; CKD, chronic kidney disease; COPD, chronic obstructive pulmonary disease; CVD, cardiovascular disease; GI, gastrointestinal; HF, heart failure; LTO, long-term outcome; MI, myocardial infarction; T2D, type 2 diabetes.

In the non-cirrhotic cohort (*n* = 12,555), key themes associated with liver-related outcomes (including cirrhosis) were chronic diseases, kidney diagnoses, cardiac diagnoses, and inpatient hospitalization Figure 4(a). Chronic diseases included anemia, CKD, diabetes, and hypertension. Kidney diagnoses comprised acute kidney failure, CKD, and hypokalemia. Cardiac diagnoses included aortic valve disorders, atrial fibrillation, coronary atherosclerosis, and heart failure.

### Long-term liver-related outcomes, excluding cirrhosis

In the cirrhotic cohort (*n* = 807), key themes associated with liver-related outcomes (excluding cirrhosis) were medications, lab tests, and injuries. The three strongest phenotypic signals were medication-related: these included hydrochlorothiazide, metformin, vitamin supplements, lisinopril, and losartan. Lab tests were generally related to metabolic deficiencies and hormones. Injuries were generally acute and included acute falls and foot sprains. However, given the sample size of the positive cohort (*n* = 86) and the lack of strong thematic coherence in these phenotypic signals, it is difficult to draw conclusions with good supporting evidence from this cohort.

### Long-term CV-related outcomes

In the non-CV cohort (*n* = 12,566), key themes associated with CV-related outcomes focused on cardiac diagnoses, kidney diagnoses, chronic diseases, and medications Figure 4(b). The arteriosclerotic disease theme most significantly presented diagnosis codes relating to coronary atherosclerosis, vessel disease of the heart, and heart failure. Other themes comprised CV diagnoses such as hypertension, atherosclerosis, and atrial fibrillation, and kidney diagnoses and chronic diseases such as diabetes, anemia, osteoarthrosis, and metabolic diagnoses. Inpatient hospitalization was also a key theme, comprising a phenotypic signal for multiple hospitalizations in combination with diagnoses for acute kidney failure and CKD.

## Healthcare costs analysis (analysis 3)

**Figure 5.**
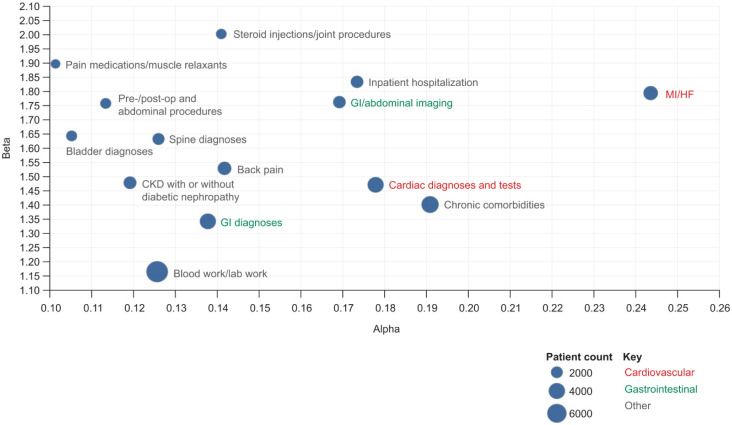
Analysis 3—Key themes associated with increased healthcare costs. CKD, chronic kidney disease; GI, gastrointestinal; HF, heart failure; MI, myocardial infarction.

One key theme associated with high healthcare costs (*n* = 10,133) comprised a grouping of heart failure procedure codes, including prothrombin time, electrocardiogram, and troponin, in addition to infection and sepsis procedure codes (Figure 5). An additional theme comprised cardiac diagnoses and testing.

A grouping of hospitalization procedure codes and kidney diagnoses was also found to have a strong phenotypic signal (Figure 5). This included both initial and subsequent hospital care, acute kidney failure, CKD, and hypokalemia. Several smaller adjacent phenotypic signals captured procedures related to hospitalization, including surgical and diagnostic procedures, and blood and lab work. Another key theme included gastrointestinal diagnoses and abdominal imaging. This theme captured patient visits, computed tomography (CT) scans of the thorax, diagnoses of splenomegaly, and similar procedure codes related to abdominal and thoracic CT scans. A smaller theme relating to gastrointestinal diagnoses was also present, containing related diagnosis codes and overlapping in theme.

## Discussion

In this retrospective cohort study, we used novel AI techniques to isolate phenotypic profiles meaningfully associated with a broad range of relevant outcomes in MASH. The phenotypic signals that emerged were generally thematically coherent, comprising information related to several aspects of patients’ histories, and the use of both alpha and beta metrics reduced the risk of missing potentially relevant signals.

In the rapid fibrosis progression analysis, CV diagnoses emerged as key themes in both the absolute (alpha) and relative (beta) phenotypic signal dimensions, highlighting an association between these diagnoses and a higher risk of rapid fibrosis progression.

In the long-term outcomes analysis, chronic diseases, kidney diagnoses, and cardiac diagnoses were positively associated with cirrhosis and other long-term liver-related outcomes in patients without cirrhosis by 6 months after MASH diagnosis, whereas medications, lab tests, and injuries were linked to additional long-term liver-related outcomes in patients who had cirrhosis by the time of MASH diagnosis. Cardiac diagnoses, kidney diagnoses, and medications were positively associated with future heart failure, myocardial infarction, stroke, or unstable angina in patients who had not experienced any of these conditions before diagnosis of MASH.

In the healthcare costs analysis, several themes associated with high costs overlapped with those in the rapid fibrosis progression analysis, including CV diagnoses, hospitalization, gastrointestinal diagnoses, and abdominal imaging. The combination of themes for heart failure, infection, and sepsis with themes for cardiac diagnoses and diagnostic testing may characterize patients who present with cardiac symptoms requiring in-depth evaluation and monitoring, leading to high healthcare resource utilization. Recent studies using health economic-focused designs report more detailed cost breakdowns,^21,22^ complementing the descriptive high‑cost patient identification approach used in the present exploratory analysis.

Although the composition of the phenotypic profiles differed across the three analyses, the strongest phenotypic signals were typically related to CV and kidney diagnoses. The emergence of CV diagnoses as a key theme associated with several outcomes may be consistent with shared risk factors observed in CVD and MASH.^23^ The key theme of kidney diagnoses may reflect the bidirectional relationship between type 2 diabetes (T2D) and MASH,^24^ and the occurrence of CKD as a comorbidity in T2D.^25^ Themes linked to diabetes were identified in the long-term clinical outcomes analysis, aligning with evidence that the presence of diabetes increases the risk of liver-related, cirrhosis-related, and all-cause mortality.^26^

Phenotypic signals were identified through an unsupervised AI-driven process based on patterns of co-occurring diagnoses, procedures, and laboratory tests, with descriptive labels assigned post hoc to summarize the dominant clinical theme. Each label is intended to capture the theme of a signal, and different clinicians might legitimately group conditions differently. However, we do our best to assign descriptive labels, with the caveat that there may be additional codes implied by the label that the algorithm has not included in the signal. For example, biomarkers such as brain natriuretic peptide and N-terminal pro-B-type natriuretic peptide were not prominent, likely reflecting limited or inconsistent measurement in routine care. Furthermore, laboratory tests, including prothrombin time, activated partial thromboplastin time, electrocardiography, and troponin, are not specific to heart failure, highlighting potential syndromic overlap and misclassification inherent in real-world analyses.

The study utilized data from several sources, and so the findings are expected to be generalizable to clinical practice. An inherent limitation of real-world data is incomplete or inconsistent medical records.^27^ However, as data are drawn from real-world healthcare settings, the insights gained are likely to be generalizable to those settings. In the future, generalizability could be assessed by using the isolated phenotypic profiles to define at-risk patient cohorts in other datasets, with subsequent evaluation of these cohorts’ outcomes after MASH diagnosis. A linked limitation is the use of ICD, MEDCIN, and SNOMED CT codes alone to identify patients with MASH, cirrhosis, other liver-related outcomes, and CV outcomes. Other measures, such as serum virologic markers, quantitative alcohol intake, and imaging or biopsy findings, were not used, which may have led to the misclassification of some patients. The sensitivity and specificity of this approach were not empirically assessed as a formal chart review, and positive predictive value assessment was not performed, but this would be an interesting direction for future research.

Despite the large population size, some cohorts and subgroups experienced significant attrition following the application of exclusion criteria, particularly in analysis 1, owing to the requirement for at least three FIB-4 scores, and the cirrhotic cohort of analysis 2, owing in part to the exclusion of patients with a FIB-4 score >2.67. Sufficient data were generally available to draw conclusions about the strengths of the identified phenotypic signals, but in several instances, a lack of data limited our ability to test signal robustness. The study eligibility criteria were selected to minimize potential bias while permitting the desired analyses; however, the requirement for survival for 6 months beyond the index for the non-cirrhotic cohort in analysis 2 meant that individuals who developed a qualifying outcome within 6 months were excluded.

The use of FIB-4 scores to define progression was based on evidence of their prognostic potential;^28^ however, additional data may be required to test the association between changes in FIB-4 scores and fibrosis stages, and further analyses could be performed using age-specific FIB-4 thresholds. Although FIB-4 is useful as an initial screening tool for identifying patients with advanced fibrosis, it has limitations in some populations, including poor specificity for advanced fibrosis in people aged ⩾65 years,^29^ and poor sensitivity in those aged <35 years and those with obesity or diabetes.^30^

Themes that are associated with inputs in the FIB-4 calculation (age, aspartate aminotransferase (AST), alanine aminotransferase (ALT), and platelet count) should be interpreted with caution owing to potential confounding effects. For example, one of the top themes for rapid fibrosis progression was thrombocytopenia, and lower platelet counts increase FIB-4 scores. Conditions such as sepsis are also associated with lower platelet counts,^31^ and hepatic congestion caused by heart failure can increase levels of AST and ALT.^32^

CV outcomes were prioritized because CVD is a leading cause of morbidity and mortality in MASLD/MASH.^6,7,23^ However, other competing outcomes that were not assessed, including CKD, extrahepatic malignancies, infections, and frailty, may also be clinically and economically relevant in this population. Quantifying the relative contribution of competing outcomes would require a dedicated survival analysis with formal competing risk modeling; this was not performed in this study, but would be an interesting topic for future research.

The results of this study demonstrate that data on patients’ health histories at the time of the first MASH diagnosis may be sufficient to separate patients into phenotypes that are meaningfully associated with different disease-burden trajectories. This suggests that multiple phenotypic elements are associated with prognosis; patients who appear to be in the same subgroup based on one factor alone may experience distinct disease trajectories due to other differences.

These findings provide a foundation for further research into predictive modeling, which could translate phenotypic signal associations into personalized assessments of burden-specific risk. This could have utility both in understanding population trajectories and for individual prediction of patients’ future disease trajectories at the time of diagnosis. In particular, the phenotypic separation of higher-risk patients into CV-dominant and liver-dominant subtypes merits further study, as these patients could potentially benefit from different management pathways starting as early as the time of their initial MASH diagnosis. Formal cost analyses to estimate the economic burden among patients with identified characteristics also represent an important direction for future work, building on the phenotypic findings reported here.

## Conclusion

This proof-of-concept study demonstrates the application of AI phenotyping to multidimensional real-world data and its ability to isolate characteristics associated with clinical and economic outcomes in MASH. A better understanding of patient characteristics associated with rapid fibrosis progression, the development of long-term clinical outcomes, and high healthcare costs could enable the identification of patients requiring more regular monitoring and suggest new approaches for management.

## Supplemental Material

sj-docx-1-tag-10.1177_17562848261462398 – Supplemental material for Using artificial intelligence to identify characteristics associated with clinical and economic outcomes in MASH (FOCUS-MASH) Supplemental material, sj-docx-1-tag-10.1177_17562848261462398 for Using artificial intelligence to identify characteristics associated with clinical and economic outcomes in MASH (FOCUS-MASH) by Kamal Kant Mangla, Semiu O. Gbadamosi, Daniel Semeniuta, Jigar Bandaria, Joseph Zabinski, Gary Curhan and Costas Boussios in Therapeutic Advances in Gastroenterology

sj-docx-2-tag-10.1177_17562848261462398 – Supplemental material for Using artificial intelligence to identify characteristics associated with clinical and economic outcomes in MASH (FOCUS-MASH) Supplemental material, sj-docx-2-tag-10.1177_17562848261462398 for Using artificial intelligence to identify characteristics associated with clinical and economic outcomes in MASH (FOCUS-MASH) by Kamal Kant Mangla, Semiu O. Gbadamosi, Daniel Semeniuta, Jigar Bandaria, Joseph Zabinski, Gary Curhan and Costas Boussios in Therapeutic Advances in Gastroenterology

sj-eps-1-tag-10.1177_17562848261462398 – Supplemental material for Using artificial intelligence to identify characteristics associated with clinical and economic outcomes in MASH (FOCUS-MASH) Supplemental material, sj-eps-1-tag-10.1177_17562848261462398 for Using artificial intelligence to identify characteristics associated with clinical and economic outcomes in MASH (FOCUS-MASH) by Kamal Kant Mangla, Semiu O. Gbadamosi, Daniel Semeniuta, Jigar Bandaria, Joseph Zabinski, Gary Curhan and Costas Boussios in Therapeutic Advances in Gastroenterology

## Declarations

**Ethics approval:** This study utilized de-identified data from an administrative claims database, thus no ethics committee approval was required.

**Consent for publication:** Not applicable.

**Availability of data and materials:** The data that support the findings of this study are available from OM1, Inc., but restrictions apply to the availability of these data, which were used under license for the current study, and so are not publicly available. Data are, however, available from the authors upon reasonable request and with permission from OM1, Inc.
