# Supplementary material for: Using artificial intelligence to identify characteristics associated with clinical and economic outcomes in MASH (FOCUS-MASH)
Source: Ther Adv Gastroenterol. 2026 Jul 9;19:17562848261462398. doi: 10.1177/17562848261462398 (PMC13351257; doi:10.1177/17562848261462398)

**Using artificial intelligence to identify characteristics associated with clinical and economic outcomes in MASH (FOCUS-MASH)**

Kamal Kant Mangla^1^, Semiu O. Gbadamosi^2^, Daniel Semeniuta^3^, Jigar Bandaria^3^, Joseph Zabinski^3^, Gary Curhan^3^, Costas Boussios^3^

^1^Novo Nordisk Service Centre India Pvt. Ltd., Bengaluru, India

^2^Novo Nordisk Inc., Plainsboro, NJ, USA

^3^OM1 Inc., Boston, MA, USA

Supplemental Material

Supplemental Table. Patient characteristics (all analyses)

| **Characteristic** | **Overall analyses 1–3**  **(N = 14,707)**^a^ | **Analysis 1: rapid fibrosis progression**  **(n = 1795)** | | **Analysis 2: long-term clinical outcomes (n = 13,880)**^b^ | | | | | | **Analysis 3: healthcare costs**  **(n = 10,133)** | |
| --- | --- | --- | --- | --- | --- | --- | --- | --- | --- | --- | --- |
|  |  |  |  | **Non-cirrhotic cohort**  **(n = 12,555)** | | **Cirrhotic cohort**  **(n = 807)** | | **Non-CV cohort**  **(n = 12,566)** | |  |  |
|  |  | **Positive  (n = 175)** | **Negative**  **(n = 1620)** | **Positive**  **(n = 313)** | **Negative**  **(n = 12,242)** | **Positive**  **(n = 86)** | **Negative**  **(n = 721)** | **Positive**  **(n = 581)** | **Negative**  **(n = 11,985)** | **Positive**  **(n = 1014)** | **Negative**  **(n = 9119)** |
| Women, n (%) | 8616 (58.6) | 114 (65.1) | 1010 (62.3) | 198 (63.3) | 7056 (57.6) | 53 (61.6) | 486 (67.4) | 369 (63.5) | 6939 (57.9) | 642 (63.3) | 5321 (58.3) |
| Age, mean (SD), years | NR^c^ | 66.3 (9.3) | 54.6 (9.1) | 57.9 (12.7) | 53.3 (13.8) | 62.0 (10.5) | 59.8 (11.9) | 61.7 (11.7) | 52.9 (13.6) | 58.4 (13.3) | 54.2 (14.1) |
| US region, n (%) | | | | | | | | | | | |
| Northeast | 2109 (14.3) | 25 (14.3) | 294 (18.1) | 29 (9.3) | 1809 (14.8) | 10 (11.6) | 88 (12.2) | 77 (13.3) | 1743 (14.5) | 141 (13.9) | 1365 (15.0) |
| Midwest | 1759 (12.0) | 15 (8.6) | 166 (10.2) | 36 (11.5) | 1484 (12.1) | 13 (15.1) | 72 (10.0) | 82 (14.1) | 1445 (12.1) | 147 (14.5) | 1167 (12.8) |
| South | 6879 (46.8) | 79 (45.1) | 763 (47.1) | 158 (50.5) | 5587 (45.6) | 46 (54.5) | 413 (57.3) | 292 (50.3) | 5439 (45.3) | 567 (55.9) | 4244 (46.5) |
| West | 3899 (26.5) | 56 (32.0) | 389 (24.0) | 88 (28.1) | 3310 (27.0) | 17 (20.0) | 145 (20.1) | 128 (22.0) | 3307 (27.6) | 156 (15.4) | 2303 (25.3) |
| Other | 23 (0.2) | 0 (0) | 3 (0.2) | 0 (0) | 22 (0.2) | 0 (0) | 0 (0) | 0 (0) | 21 (0.2) | 1 (0.1) | 17 (0.2) |
| Unknown | 38 (0.3) | 0 (0) | 5 (0.3) | 2 (0.6) | 30 (0.2) | 0 (0) | 3 (0.4) | 2 (0.3) | 30 (0.3) | 2 (0.2) | 23 (0.3) |

^a^Some patients featured in more than one analysis, so the total is lower than the sum of the total for each analysis. ^b^Some patients featured in more than one cohort, so the total is lower than the sum of the total for each cohort. ^c^Patients do not have a consistent index date across analyses, so age cannot be reported for the study cohort as a whole.

CV, cardiovascular; NR, not reported; SD, standard deviation.

**Supplemental Figure**. Overview of modeling process.


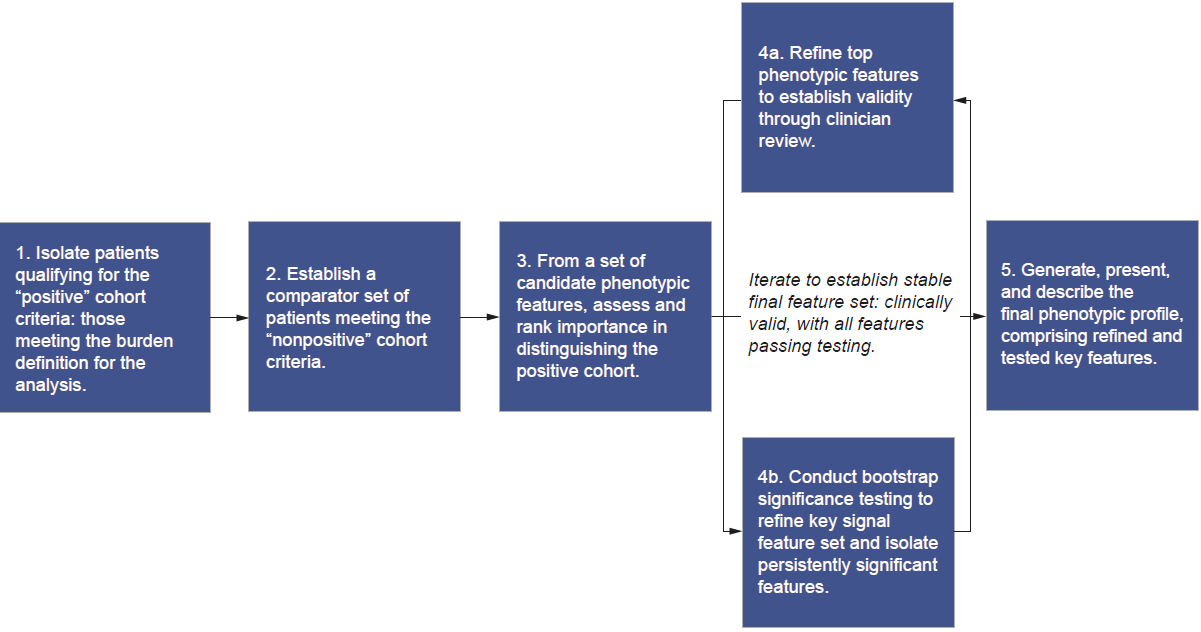

Supplement: sj-docx-2-tag-10.1177_17562848261462398 – Supplemental material for Using artificial intelligence to identify characteristics associated with clinical and economic outcomes in MASH (FOCUS-MASH) [file sj-docx-2-tag-10.1177_17562848261462398.docx]
